# Intracellular complexes of the early-onset torsion dystonia-associated AAA+ ATPase TorsinA

**DOI:** 10.1186/2193-1801-3-743

**Published:** 2014-12-16

**Authors:** Hui Li, Hui-Chuan Wu, Zhonghua Liu, Lucia F Zacchi, Jeffrey L Brodsky, Michal Zolkiewski

**Affiliations:** Department of Biochemistry and Molecular Biophysics, Kansas State University, Manhattan, KS 66506 USA; Department of Biological Sciences, University of Pittsburgh, Pittsburgh, PA 15260 USA; Department of Embryology, Carnegie Institution, Baltimore, MD 21218 USA

**Keywords:** Early-onset dystonia, TorsinA, AAA+ ATPase, Protein association

## Abstract

A single GAG codon deletion in the gene encoding torsinA is linked to most cases of early-onset torsion dystonia. TorsinA is an ER-localized membrane-associated ATPase from the AAA+ superfamily with an unknown biological function. We investigated the formation of oligomeric complexes of torsinA in cultured mammalian cells and found that wild type torsinA associates into a complex with a molecular weight consistent with that of a homohexamer. Interestingly, the dystonia-linked variant torsinAΔE displayed a reduced propensity to form the oligomers compared to the wild type protein. We also discovered that the deletion of the N-terminal membrane-associating region of torsinA abolished oligomer formation. Our results demonstrate that the dystonia-linked mutation in the torsinA gene produces a protein variant that is deficient in maintaining its oligomeric state and suggest that ER membrane association is required to stabilize the torsinA complex.

**Electronic supplementary material:**

The online version of this article (doi:10.1186/2193-1801-3-743) contains supplementary material, which is available to authorized users.

## Background

Early-onset torsion dystonia (EOTD) is the most common and severe form of primary dystonia, a neurological disorder that manifests as uncontrollable movements and abnormal body postures. Most cases of EOTD are associated with a deletion of a single GAG codon in the *DYT1* gene. As a result, a single glutamic acid residue is absent in the EE pair located in the C-terminal region of torsinA (Ozelius et al. 1997). TorsinA is a putative member of the AAA+ superfamily of *A* TPases *a* ssociated with different *a* ctivities (Neuwald et al. 1999). The torsinA mRNA is widely expressed in various human tissues, including the central nervous system, but the biological role of torsinA is not completely clear (reviewed in (Tanabe et al. 2009; Zolkiewski and Wu 2011)). AAA+ ATPases are energy-driven “molecular machines”, which remodel the conformation of macromolecules and disassemble macromolecular complexes (Hanson and Whiteheart 2005). Proteins from the AAA+ family form ring-shaped hexameric complexes, which enclose their substrate molecules. Hexamer formation is essential for the activity of AAA+ ATPases (Barnett et al. 2000). Numerous torsinA partners have been identified and the association with some of these is compromised when the mutant gene product is expressed (Naismith et al. 2009; Zolkiewski and Wu 2011). However, the identity of a torsinA substrate that is critical for its cellular activity is unknown and, more fundamentally, whether torsinA even forms a hexamer in cells has not been fully established. Moreover, it is unclear how the glutamate deletion affects these biochemical properties and in turn, which defect(s) associated with the mutant protein are linked to EOTD.

The torsinA sequence contains an N-terminal ER-targeting signal peptide that is cleaved upon import into the ER lumen, producing the mature 36-kDa form of the protein (Liu et al. 2003). The signal sequence is followed by a 20-residue-long hydrophobic segment that is responsible for membrane association (Liu et al. 2003) and ER retention (Vander Heyden et al. 2011). The AAA+ module of torsinA is located downstream of the membrane-binding domain and contains a non-canonical ATP-binding Walker-A motif (Nagy et al. 2009; Zolkiewski and Wu 2011) and six cysteines that are absent from other AAA+ ATPases (Zhu et al. 2008; Zolkiewski and Wu 2011). The site of the dystonia-linked glutamate deletion (E302/E303) is located within the C-terminal AAA+ subdomain, which supports oligomerization of other AAA+ ATPases (Barnett et al. 2000). Studies with purified recombinant torsinA revealed either a monomeric protein (Kustedjo et al. 2003; Zhu et al. 2008) or a spectrum of high-molecular weight particles (Zhao et al. 2013). In contrast, torsinA assemblies ranging from monomers and dimers to hexamers were detected in lysates from mammalian cells (Kustedjo et al. 2000; Gordon and Gonzalez-Alegre 2008; Vander Heyden et al. 2009; Jungwirth et al. 2010). How these assemblies are affected by the disease-causing mutation or the hydrophobic membrane anchor has not yet been established.

To this end, we investigated the size of human torsinA complexes after isolation from cultured mammalian cells. We found that the main oligomeric species is consistent with the formation of torsinA hexamers, but this structure becomes less stable when the dystonia-linked protein variant is expressed. We also found that the membrane-bound hydrophobic segment stabilizes the torsinA oligomer. These data add fundamental new insights to our understanding of torsinA structure and suggest why the loss of a single amino acid can exhibit profound cellular effects.

## Results and discussion

**Figure 1:**
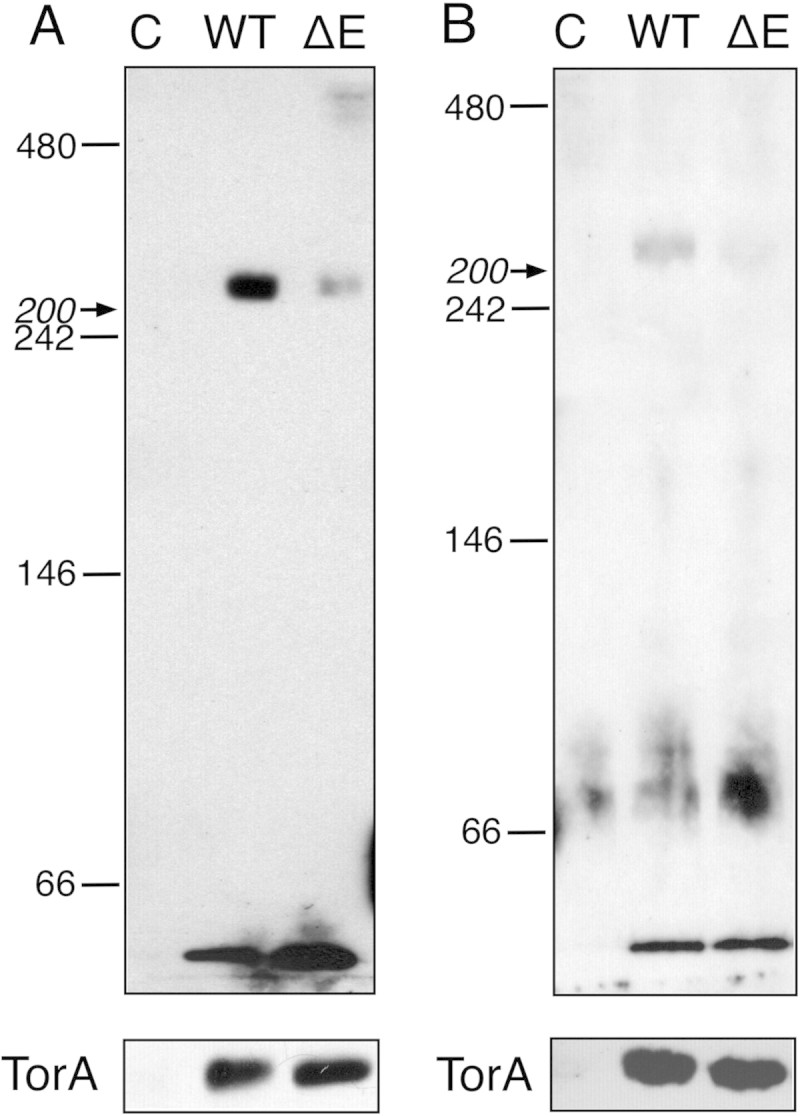
**BN-PAGE analysis of torsinA complexes.** Full-length human torsinA (WT) or the dystonia-linked torsinAΔE protein (ΔE) was expressed in HEK293 **(A)** and CHO **(B)** cells. Production of the torsinA variants was confirmed by SDS-PAGE followed by immunoblotting with anti-torsinA antibodies (lower panels) using untransfected cells as a control (C). The cell lysates were separated on BN-PAGE followed by immunoblotting (upper panels). For BN-PAGE, the migration positions of the native-electrophoresis standards are indicated. The migration position of β-amylase (200 kDa) is indicated with an arrow. Protein migration in BN-PAGE can reflect other biophysical properties, besides the molecular weight, so the molecular weight determination is only approximate. The figure shows a representative result from two independent experiments.

To determine whether human torsinA and the dystonia-linked torsinAΔE variant oligomerize in the cell, we expressed each protein in two cell lines, HEK293 and CHO cells. After preparation of cell lysates in dodecylmaltoside, BN-PAGE and immunoblotting with an anti-torsinA antibody was used to observe the distribution of the torsinA-containing species (Figure 1). Both stably transfected cell lines produced comparable amounts of torsinA and torsinAΔE (Figure 1A, B, lower panels). In addition to some monomeric torsinA and torsinAΔE (shown by the bands below 66 kDa), BN-PAGE detected a single major immunoreactive species migrating close to the 200-kDa complex of β-amylase, but slower than the 242-kDa protein standard (Figure 1A, B, upper panels). The migration of the torsinA oligomer in BN-PAGE is consistent with that of a homohexamer (predicted molecular weight 216 kDa) and is consistent with the formation of a species of similar size in BN-PAGE using lysates prepared from U2OS cells (Vander Heyden et al. 2009). It cannot be excluded, however, that the detected species corresponds to a hetero-oligomer containing torsinA and other components, such as the torsinA binding partners LAP1 and LULL1 (Goodchild and Dauer 2005; Zhao et al. 2013; Sosa et al. 2014). We also found that the deletion of Glu302 in torsinA apparently destabilizes the oligomeric species (Figure 1A, B, upper panels). This result suggests that the dystonia-linked torsinA variant may be defective in either self-association or interactions with other proteins. Indeed, the efficiency of torsinAΔE interaction with LAP1 and LULL1 is compromised relative to the wild type protein (Naismith et al. 2009; Zhao et al. 2013). In contrast, the dystonia-linked torsinAΔE variant shows an enhanced binding affinity for nesprin (Nery et al. 2008). Thus, the apparent loss of the detected oligomeric species in the torsinAΔE producing cells (Figure 1) suggests that the observed torsinA complex does not include nesprin. Nevertheless, the data presented in Figure 1 indicate that the EOTD-associated mutation has a profound effect on oligomer and/or complex formation, and we propose that this defect might impact the protein’s function and disease presentation.

**Figure 2:**
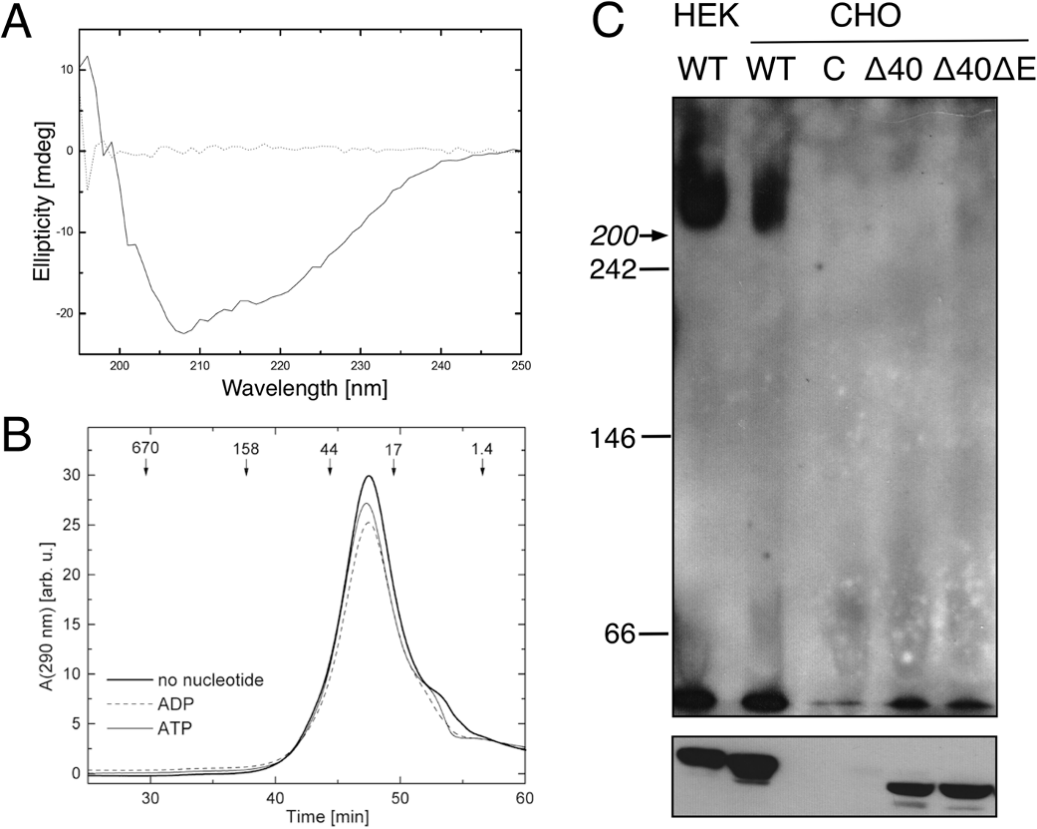
**Oligomerization of the N-terminally truncated torsinA variants. (A)** Far-UV circular dichroism spectra of purified torsinAΔ40 (1 mg/ml, solid line) and the dialysis buffer (dotted line) are shown. **(B)** Gel-filtration analysis of torsinAΔ40 in the absence of nucleotides or in the presence of 2 mM ATP or ADP is shown. The elution times of molecular weight standards (kDa) are indicated. **(C)** BN-PAGE (upper panel) and SDS-PAGE (lower panel) analysis was followed by immunoblotting with anti-torsinA antibodies of lysates from HEK293 and CHO cells expressing either full-length torsinA (WT), torsinAΔ40 (Δ40), torsinAΔ40ΔE (Δ40ΔE) or untransfected cells (C). For BN-PAGE, the migration positions of the native-electrophoresis standards are indicated. The migration position of β-amylase (200 kDa) is indicated with an arrow. Protein migration in BN-PAGE can reflect other biophysical properties, besides the molecular weight, so the molecular weight determination is only approximate. The figure shows a representative result from two independent experiments.

As noted above, previous studies on purified full-length torsinA failed to detect oligomeric species, which could have been the result of detergent-induced destabilization of intersubunit contacts (Kustedjo et al. 2003). Other experiments using a purified protein detected higher molecular weight species that were not further resolved (Zhao et al. 2013). To obtain enriched soluble protein in the absence of a detergent, we produced a truncated torsinA variant lacking the hydrophobic membrane-binding region, torsinAΔ40 (Liu et al. 2003). TorsinAΔ40 was expressed in S2 cells and purified from the culture media (see Materials and Methods). Interestingly, torsinAΔ40ΔE was poorly secreted in S2 culture (Liu et al. 2003), which is consistent with the apparent mislocalization of this dystonia variant from the ER lumen to the nuclear envelope (Goodchild and Dauer 2004; Naismith et al. 2004). The circular dichroism spectrum of torsinAΔ40 (Figure 2A) was similar to that of the full-length torsinA purified with detergent (Kustedjo et al. 2003), which indicates that a deletion of the hydrophobic segment does not inhibit folding of torsinA. TorsinAΔ40 was strictly monomeric (~30 kDa, Figure 2B), regardless of whether size exclusion chromatography was run in the absence of nucleotides or in the presence of ATP or ADP, which in other cases stabilize AAA+ hexamers (Akoev et al. 2004).

To corroborate these data, we next investigated the oligomeric state of torsinAΔ40 in mammalian cell lysates (Figure 2C). In contrast to the full-length protein (WT), the torsinAΔ40 and torsinAΔ40ΔE variants again failed to form oligomeric species in BN-PAGE. This result is in accordance with the properties of purified torsinAΔ40 (Figure 2B) and indicates that the 20 residue-long N-terminal hydrophobic segment is essential to stabilize torsinA complexes. Two mechanisms can be proposed to account for this result. First, the hydrophobic segment may directly participate in either self-association or hetero-association with another protein’s membrane-embedded domain. Second, the membrane association of torsinA and its retention in the ER lumen may increase the likelihood of forming the homo- or heterooligomers. Recently, hetero-hexamers of torsinA and LAP1 were reconstituted with purified proteins (Sosa et al. 2014). Since LAP1 is a transmembrane protein, its interaction with torsinA in the cell might be efficient only if torsinA is also targeted to the ER membrane by its N-terminal hydrophobic segment. Future efforts will be directed to address this hypothesis.

## Conclusions

In summary, we found that torsinA forms a discrete high-molecular weight complex in mammalian cells. However, the complex is destabilized by the dystonia-linked mutation, but is stabilized by the membrane anchor. Establishing a link between defects in torsinAΔE oligomerization and EOTD will be an important focus of future research efforts.

## Methods

### Plasmids, antibodies, and reagents

DNA constructs containing the human torsinA sequence in pcDNA3 vector were described before (Liu et al. 2003) and anti-torsin antibodies were obtained as described (Zacchi et al. 2014). Sweet potato β-amylase was from Sigma. Native electrophoresis protein standards were from Invitrogen/Life Technologies.

### Cell culture

HEK293 cells were maintained in DMEM (Invitrogen) supplemented with 10% fetal bovine serum (BioWhittaker) at 37°C in the presence of 5% CO_2_. CHO-K1 cells (ATCC) were maintained in F-12 K medium (Invitrogen) supplemented with 10% fetal bovine serum. Cells were transfected with pcDNA3 expression vectors containing the torsinA variants using FuGene 6 transfection reagent (Roche) according to the manufacturer’s instructions. Stably transfected cells were selected in the presence of 1 mg/mL G418 (Invitrogen).

### Blue-native PAGE

BN-PAGE is a native gel electrophoresis technique, where the Coomassie Brilliant Blue dye binds to membrane protein complexes and provides the electric charge for the electrophoretic separation. BN-PAGE was carried out as previously described (Wittig et al. 2006; Vander Heyden et al. 2009; Jungwirth et al. 2010). Briefly, ~90% confluent cells were collected and solubilized in a lysis buffer (50 mM imidazole pH 7.0, 50 mM NaCl, 2 mM 6-aminohexanoic acid, 4 mM MgCl_2_, 2 mM EDTA, 2 mM ATP, 1 mM PMSF, and 0.25% dodecylmaltoside) for 15 min in 4°C, and centrifuged twice for 15 min at 15,000 rpm in IEC Micromax benchtop centrifuge. The supernatant supplemented with 0.0625% Coomassie blue G-250 and 5% glycerol was loaded onto a 9% polyacrylamide gel. Following electrophoresis, separated proteins were transferred onto PVDF membrane and subjected to immunoblotting using anti-torsinA antibodies, followed by horseradish peroxidase-conjugated anti-rabbit IgG antibodies (Southern Biotechnology). Signal detection was performed with WestPico chemiluminescence kit (Pierce).

### Protein purification

The S2 cell line stably transfected with a torsinAΔ40 expression plasmid containing the BiP signal sequence followed by an N-terminal His-tag was produced as previously described (Liu et al. 2003). Cells were grown at 23°C in S2 medium (Invitrogen) supplemented with 0.5% DMSO when the density reached 10^7^ cells/ml and protein expression was induced after 24 h with 0.7 mM CuSO_4_. The cells were separated from the culture medium by centrifugation 6 days post induction. The medium was filtered through a 0.45 μm membrane and loaded onto a 2.5-ml Chelating Sepharose column (GE Healthcare). Proteins were eluted with an imidazole step concentration gradient. Fractions containing torsinAΔ40 (eluted at 10–50 mM imidazole) were pooled, concentrated on Centriplus YM-10 (Millipore), and dialyzed in 50 mM Tris–HCl pH 7.5, 100 mM NaCl, 20 mM MgCl_2_, and 10% glycerol.

### Circular dichroism spectroscopy

CD spectra were measured with a Jasco J-720 spectrometer using a 0.01-cm cylindrical cuvette at room temperature.

## Gel filtration chromatography

Gel filtration analysis was performed at room temperature with a Shimadzu HPLC. TorsinAΔ40 samples (20 μl, ~0.2 mg/ml) were analyzed at 0.04 ml/min on a Superdex 200 PC 3.2/30 column (GE Healthcare) equilibrated in 50 mM Tris/HCl pH 7.5, 0.2 M KCl, 20 mM MgCl_2_ without nucleotides or with 2 mM ATP or ADP.

## Authors’ original submitted files for images

Below are the links to the authors’ original submitted files for images. Authors’ original file for figure 1 Authors’ original file for figure 2
