# Supplementary figures and images for: Intracellular complexes of the early-onset torsion dystonia-associated AAA+ ATPase TorsinA
Source: Springerplus. 2014 Dec 16;3:743. doi: 10.1186/2193-1801-3-743 (PMC4320221; doi:10.1186/2193-1801-3-743)

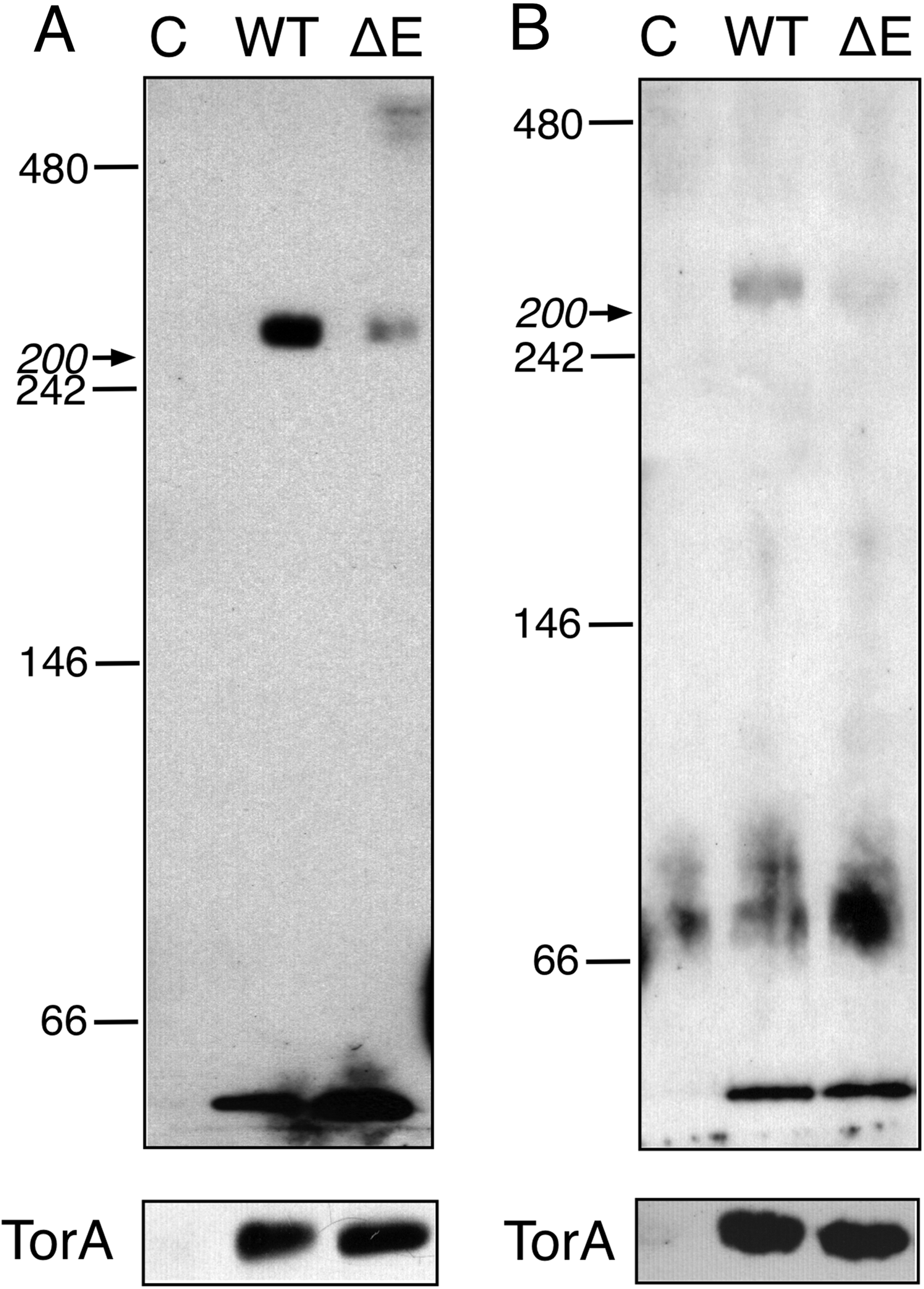

Supplement: Supplementary file 1 — Authors’ original file for figure 1 [file 40064_2014_1493_MOESM1_ESM.tif]

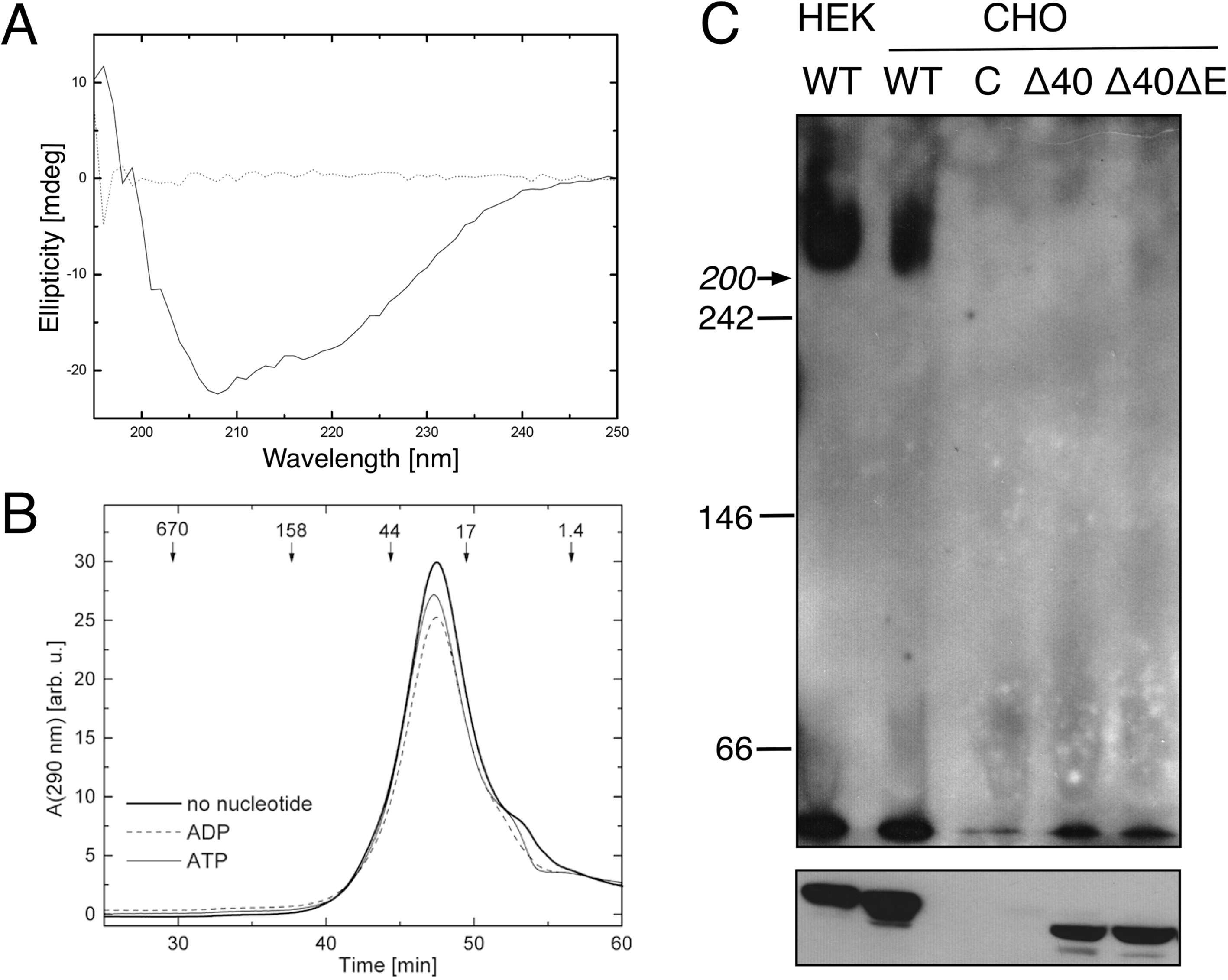

Supplement: Supplementary file 2 — Authors’ original file for figure 2 [file 40064_2014_1493_MOESM2_ESM.tif]
